# Quantitative magnetic resonance imaging (MRI) evaluation of visceral and hepatic fat in Cushing’s syndrome: insights into cortisol-driven metabolic changes

**DOI:** 10.20945/2359-4292-2026-0109

**Published:** 2026-09-11

**Authors:** Rania Naguib, Marwa Ahmed Salah Ahmed, Sherif Mohamed Elsharkawi, Hany A. Elkattawy, Mohamed Ahmed Hassan, Hend Naguib

**Affiliations:** 1 Department of Internal Medicine, College of Medicine, Princess Nourah bint Abdulrahman University, Riyadh, Saudi Arabia; 2 Department of Internal Medicine, Endocrinology Unit, Faculty of Medicine, Alexandria University, Alexandria, Egypt; 3 Department of Clinical and Chemical Pathology, Faculty of Medicine, Alexandria University, Alexandria, Egypt; 4 Department of Basic Medical Sciences, College of Medicine, AlMaarefa University, Diriyah, Riyadh, Saudi Arabia; 5 Deanship of Scientific Research and Post-Graduate Studies, AlMaarefa University, Diriyah, Riyadh, Saudi Arabia; 6 Department of Radiology, Faculty of Medicine, Zagazig University, Zagazig, Sharkia, Egypt; 7 Department of Internal Medicine, Hepatology Unit, Faculty of Medicine, Alexandria University, Alexandria, Egypt

**Keywords:** Magnetic resonance imaging (MRI), Cushing’s syndrome, hepatic steatosis, visceral fat, cardiometabolic

## Abstract

**Objective:**

To quantify hepatic steatosis and visceral adiposity in active Cushing’s syndrome (CS) using magnetic resonance imaging (MRI), evaluate associations between hypercortisolism and MRI-derived fat metrics, and characterize the relationship between hepatic and visceral fat compartments.

**Subjects and methods:**

This retrospective cross-sectional study assessed 36 patients with biochemically confirmed active CS. Hepatic proton density, fat fraction, and visceral adipose tissue area were quantified using chemical-shift encoded MRI with semi-automated segmentation. Biochemical assessments included serum cortisol, adrenocorticotropic hormone, liver enzymes, and lipid profiles.

**Results:**

MRI-defined hepatic steatosis (fat fraction ≥6.25%) was identified in 69.4% (25/36) of patients. Those with steatosis exhibited higher serum cortisol levels (832.9 ± 347.8 vs. 581.2 ± 331.9 nmol/L, *p* = 0.049) and greater visceral adiposity (35.0 ± 8.0 vs. 23.9 ± 6.7 cm^2^, *p* < 0.001). Serum cortisol positively correlated with visceral fat area (*r* = 0.512, *p* = 0.001) and inversely correlated with liver signal intensity index (*r* = -0.424, *p* = 0.01). Visceral adiposity demonstrated a strong positive correlation with hepatic steatosis grade (*r* = 0.601, *p* < 0.001).

**Conclusion:**

Hepatic steatosis was highly prevalent in active CS and closely linked to hypercortisolism and visceral adiposity. These findings support a mechanistic coupling between central and ectopic fat accumulation. Integrated MRI-based fat quantification provides a non-invasive, radiation-free approach for refined cardiometabolic risk stratification in this high-risk population.

## INTRODUCTION

Endogenous Cushing’s syndrome (CS) is a state of chronic glucocorticoid excess that confers a significant metabolic burden, characterized by visceral adiposity, insulin resistance, diabetes mellitus, atherogenic dyslipidemia, and hypertension (^1^). Persistent elevations of cortisol promote hepatic gluconeogenesis and impair insulin sensitivity, while also altering both distribution and function of peripheral adipose tissue. Collectively, these effects create an environment conducive to both direct and indirect hepatic triglyceride deposition (^2^).

In parallel, the conceptual framework for fatty liver disease has evolved. The term non-alcoholic fatty liver disease has been replaced by metabolic dysfunction-associated steatotic liver disease (MASLD), defined by imagingor histology-confirmed hepatic steatosis in the presence of at least one of five cardiometabolic risk factors, in the absence of significant alcohol consumption or other chronic liver disorders (^3^). Within this context, hepatic steatosis is regarded as the hepatic manifestation of the metabolic syndrome (^4^). Glucocorticoid excess contributes to MASLD pathogenesis through several convergent mechanisms: (a) expansion of visceral adipose tissue (VAT) with increased portal delivery of free fatty acids; (b) glucocorticoid receptor (GR)-mediated upregulation of hepatic lipogenic enzymes; (c) suppression of fatty acid oxidation; and (d) aggravation of systemic insulin resistance (^5^).

Although histological assessment of liver tissue remains the reference standard for diagnosing hepatic steatosis, liver biopsy is invasive, carries a non-negligible risk of bleeding and sampling variability, and is impractical for screening or large-scale epidemiological research (^6^). Consequently, non-invasive imaging has become central to clinical practice and research. Recent MRI techniques allow precise, non-invasive quantification of visceral and hepatic fat, improving diagnosis of metabolic syndrome and MASLD. These methods aim to deepen our understanding of the relationship between hepatic and visceral fat and obesity-related conditions such as metabolic syndrome, MASLD, and cardiovascular disease (^7^).

Chemical shift-encoded magnetic resonance imaging (MRI), using in-phase and opposed-phase acquisitions, enables accurate and reproducible quantification of hepatic fat by exploiting the resonance frequency differences between water and fat protons. Proton magnetic resonance spectroscopy offers even greater precision, although its use is primarily confined to specialized research settings due to technical complexity and limited accessibility (^8^). In contrast, most previous research on body fat distribution in CS employed computed tomography (CT) (^9^). Although CT provides excellent spatial resolution for adipose tissue mapping, it involves ionizing radiation, which is an essential concern in metabolically compromised populations, and is insufficiently sensitive to detect mild hepatic steatosis, particularly when fat content is below approximately 30% (^10^).

Various imaging modalities have been employed to assess body composition in CS, including whole-body MRI (^11^), dual-energy X-ray absorptiometry (^12^), magnetic resonance spectroscopy for marrow and intrahepatic lipids assessment (^13^), and CT (^14^).

This study introduces several methodological strengths and contemporary innovations. First, it uses MRI as a single, radiation-free modality to simultaneously obtain quantitative measurements of hepatic steatosis and visceral fat area in patients with active CS. Second, it incorporates a dedicated semi-automated segmentation platform (SliceOmatic V5.0) for precise visceral fat quantification, a technique not previously described in the CS literature. Third, it directly examines associations between circulating cortisol concentrations and high-resolution MRI-derived metabolic indices, offering granular links between biochemical hypercortisolism and organ-level fat phenotypes.

Accordingly, this study aimed to establish the prevalence and severity of hepatic steatosis (MASLD) in active CS using MRI-based quantification, examine associations between serum cortisol levels and MRI-derived markers of adiposity and steatosis, and characterize the relationship between visceral fat burden and hepatic steatosis grade.

## SUBJECTS AND METHODS

### Study design and population

This cross-sectional study retrospectively analyzed data from 36 consecutive patients with newly diagnosed, treatment-naïve active CS who were managed at Alexandria University Hospital between January 2023 and January 2025. The diagnosis of CS followed current Endocrine Society Clinical Practice Guidelines (^15^), requiring: (a) elevated midnight serum cortisol (>50 nmol/L) during sleep; (b) failure to suppress serum cortisol to <50 nmol/L at 48 h after administration of dexamethasone 0.5 mg every 6 h for 48 h; and (c) confirmatory dynamic testing consistent with either adrenocorticotropic hormone (ACTH)-dependent or ACTH-independent etiology.

### Exclusion criteria

Patients were excluded if they met any of the following criteria: (a) known chronic liver disease of any cause, including viral hepatitis (hepatitis B surface antigen or hepatitis C antibody positive), autoimmune liver disease, drug-induced liver injury, cholestatic liver disease, alcohol-related liver disease (≥20 g/day for women, ≥30 g/day for men), or hereditary hemochromatosis; (b) previous treatment with any cortisol-lowering agents (ketoconazole, metyrapone, osilodrostat, or mitotane) for >4 weeks; (c) standard contraindications to MRI (ferromagnetic implants, claustrophobia, or pregnancy); or (d) age <18 years.

### Clinical and anthropometric assessment

All patients underwent comprehensive medical history reviews and physical examinations. Body mass index (BMI) was calculated as weight (kg) divided by height (m) squared. Waist circumference was measured at the midpoint between the lower costal margin and the iliac crest.

### Biochemical analyses

Fasting venous blood samples were collected from the antecubital vein between 8:00-9:00 am following an overnight fast of at least 10 h. All biochemical analyses were performed within 4 h of collection. Serum cortisol was measured using an electrochemiluminescence immunoassay (Cobas e601, Roche Diagnostics, Germany). The diurnal serum cortisol profile was calculated as the mean of five samples collected at 9:00 am, 12:00 pm, 5:00 pm, 9:00 pm, and 12:00 am. Plasma ACTH and serum dehydroepiandrosterone sulfate were measured using chemiluminescent immunometric assays (Immulite 2000, Siemens Healthcare Diagnostics, Germany). Additional laboratory assessments included alanine aminotransferase (ALT), aspartate aminotransferase (AST), gamma-glutamyl transferase (GGT), alkaline phosphatase (ALP), total bilirubin, fasting plasma glucose, total cholesterol, triglycerides, high-density lipoprotein cholesterol (HDL-C), low-density lipoprotein cholesterol (LDL-C), and serum uric acid. All biochemical assays were performed using standard enzymatic colorimetric methods on an automated analyzer (AU680, Beckman Coulter, USA).

### Magnetic resonance imaging acquisition

All imaging was performed using a 1.5-Tesla scanner (MAGNETOM Aera, Siemens Healthineers, Germany) with an 18-channel body matrix coil and 32-channel spine matrix coils. Patients were scanned in the supine position with arms elevated above the head to minimize wraparound artifacts.

### Hepatic fat quantification

A breath-hold three-dimensional T1-weighted dual-echo Dixon volumetric interpolated breath-hold examination sequence was acquired in the axial plane during a single 18-second breath-hold. Acquisition parameters were: repetition time of 7.0 ms; echo time 1 2.38 ms (opposed-phase); echo time 2 4.76 ms (in-phase); flip angle 10°; slice thickness 3.0 mm; intersection gap 0 mm; matrix 256 × 192; field of view 380 × 285 mm^2^; voxel size 1.5 × 1.5 × 3.0 mm; and parallel imaging acceleration factor 2. Fat-only, water-only, in-phase, and opposed-phase images were automatically reconstructed by the scanner software.

### Visceral fat quantification

Axial T1-weighted fast spin-echo sequences without fat saturation were acquired through the abdomen, centered at the L4-L5 intervertebral disc level. Acquisition parameters included: repetition time of 400 ms; TE 10 ms; echo train length 3; slice thickness 8.0 mm; intersection gap 2.0 mm; matrix 320 × 224; field of view 400 × 300 mm^2^; number of signal averages 2; and total acquisition time of 2 minutes and 14 seconds.

### Image analysis

All image analyses were performed by a single radiologist with 12 years of experience in abdominal imaging, who was blinded to all clinical and biochemical data. Intra-observer reproducibility was assessed by reanalyzing 10 randomly selected studies after a 4-week interval.

### Hepatic steatosis quantification

Analysis was conducted using dedicated post-processing software (Syngo.via, Siemens Healthineers, Germany). Three circular regions of interest (ROIs) of 2.0 cm^2^ each were placed in the right hepatic lobe (Couinaud segments V-VIII) on both opposed-phase and corresponding in-phase images. ROIs were carefully positioned to exclude hepatic vessels, bile ducts, the liver capsule, and artifacts. The mean signal intensity (SI) from the three ROIs was calculated. The hepatic fat fraction was then calculated using **Equation 1:**


(1) Fat fraction (%)=[(SI_in-phase - SI_opposed-phase) / (2 ×
SI_in-phase)]×100

Hepatic steatosis was defined as a hepatic fat fraction ≥6.25% based on the 95^th^ percentile of hepatic triglyceride content in healthy, normal-weight populations, as determined by proton magnetic resonance spectroscopy (^16^).

### Visceral fat quantification

Visceral fat area was quantified using semi-automated segmentation software (SliceOmatic V5.0, Tomovision, Canada). This study quantified VAT on a single axial slice at the L4-L5 level, a validated and widely adopted approach for total visceral fat estimation in large-scale studies due to its simplicity, reproducibility, and feasibility compared to multi-slice volumetric approaches. On the axial T1-weighted image at the L4-L5 intervertebral disc level, the visceral fat compartment was defined by manually tracing the inner aspect of the abdominal wall musculature (transversus abdominis, internal oblique, external oblique, and rectus abdominis) and the anterior border of the vertebral body. Pixels within the defined compartment falling within the fat intensity range (as determined by histogram analysis) were automatically segmented and quantified. Visceral fat area was expressed as total surface area in square centimeters (cm^2^).

### Statistical analysis

Statistical analyses were performed using SPSS v. 25.0 (IBM Corp., USA). The normality of continuous variables was assessed with the Shapiro-Wilk test and visual inspection of Q-Q plots. Continuous variables are presented as mean ± standard deviation for normally distributed data, or as median with interquartile range for non-normally distributed data. Categorical variables are described as absolute counts and percentages. For comparative analyses, patients were stratified into two groups according to hepatic steatosis status, defined by an MRI-derived fat fraction ≥6.25%. This a priori grouping enabled identification of clinical, biochemical, and anthropometric factors associated with hepatic steatosis in active CS. Comparisons between groups were carried out using the independent samples t-test for normally distributed continuous variables and the Mann-Whitney U test for non-normally distributed variables. Categorical variables were compared using Chi-square or Fisher’s exact test, as applicable. Correlations were assessed by Pearson’s correlation coefficient for normally distributed variables and Spearman’s rank correlation coefficient otherwise. Multiple linear regression was originally planned to identify independent predictors of hepatic fat fraction; however, to avoid model overfitting due to small sample size, analyses were limited to bivariate correlations. A two-tailed *p*-value < 0.05 was regarded as statistically significant for all analyses.

## RESULTS

### Patient characteristics and group stratification

The study cohort comprised 36 patients with active, treatment-naïve CS. The mean age was 50.2 ± 13.8 years, most participants were female (29/36, 80.6%), the mean BMI was 28.0 ± 7.0 kg/m^2^ and impaired glucose tolerance or type 2 diabetes mellitus was present in 15 (41.7%) patients (**Table 1**). Using the predefined MRI-derived hepatic fat fraction threshold of ≥6.25%, patients were stratified into two groups: those without hepatic steatosis (n = 11, 30.6%) and those with hepatic steatosis (n = 25, 69.4%). This stratification enabled identification of factors associated with hepatic steatosis in active CS and comparison of metabolic and imaging parameters between these clinically distinct phenotypes.

**Table 1:** Demographic, biochemical, and MRI characteristics stratified by hepatic steatosis status

Parameter	Total (n = 36)	Steatosis		*p*-value
		Non-hepatic (n = 11)	Hepatic (n = 25)	
Demographics				
Age, years	50.2 ± 13.8	44.0 ± 15.8	55.1 ± 11.9	0.03
Female sex, n (%)	29 (80.6)	9 (81.8)	20 (80.0)	0.80
BMI, kg/m^2^	28.0 ± 7.0	24.0 ± 5.0	29.6 ± 7.7	0.03
Biochemistry				
Serum cortisol, nmol/L	755.9 ± 364.2	581.2 ± 331.9	832.9 ± 347.8	0.049
Total cholesterol, mmol/L	7.45 ± 2.44	6.22 ± 1.45	7.99 ± 2.71	0.01
Triglycerides, mmol/L	1.76 ± 1.01	1.22 ± 0.65	2.00 ± 1.09	0.01
LDL-C, mmol/L	5.13 ± 1.72	4.48 ± 1.18	5.42 ± 1.87	0.02
HDL-C, mmol/L	1.34 ± 0.23	1.46 ± 0.15	1.29 ± 0.24	0.05
ALT, U/L	45.3 ± 30.6	30.0 ± 14.9	52.0 ± 34.1	0.049
AST, U/L	24.9 ± 10.0	20.0 ± 10.2	27.0 ± 9.1	0.048
GGT, U/L	39.2 ± 10.1	34.6 ± 8.2	41.2 ± 10.1	0.045
Total bilirubin, µmol/L	9.9 ± 5.7	7.0 ± 2.8	11.2 ± 6.4	0.045
Impaired glucose/diabetes (%)	15 (41.7)	4 (36.4)	11 (44.0)	0.29
Hyperuricemia, n (%)	6 (16.7)	2 (18.2)	4 (16.0)	0.76
MRI metrics				
Hepatic fat fraction, %	25.2 ± 5.8	5.40 ± 1.51	45.0 ± 10.1	<0.001
Visceral fat area, cm^2^	31.6 ± 9.6	23.9 ± 6.7	35.0 ± 8.0	<0.001

Data presented as mean ± standard deviation.

### Demographic and anthropometric comparisons

Patients with hepatic steatosis were significantly older than those without steatosis (55.1 ± 11.9 vs. 44.0 ± 15.8 years, *p =* 0.03). BMI was also significantly higher in the steatosis group (29.6 ± 7.7 vs. 24.0 ± 5.0 kg/m^2^, *p =* 0.03). No significant difference in sex distribution was observed between groups (*p =* 0.80) (**Table 1**).

### Biochemical comparisons

As shown in **Table 1**, patients with hepatic steatosis had significantly higher serum cortisol concentrations than those without steatosis (832.9 ± 347.8 vs. 581.2 ± 331.9 nmol/L, *p =* 0.049). The lipid profile was significantly more atherogenic in the steatosis group, with higher total cholesterol (7.99 ± 2.71 vs. 6.22 ± 1.45 mmol/L, *p =* 0.01), triglycerides (2.00 ± 1.09 vs. 1.22 ± 0.65 mmol/L, *p =* 0.01), and LDL-C (5.42 ± 1.87 vs. 4.48 ± 1.18 mmol/L, *p =* 0.02). HDL-C was lower in the steatosis group, approaching but not reaching statistical significance (1.29 ± 0.24 vs. 1.46 ± 0.15 mmol/L, *p =* 0.05). Markers of hepatocellular injury were significantly elevated in patients with hepatic steatosis: ALT (52.0 ± 34.1 vs. 30.0 ± 14.9 U/L, *p =* 0.049), AST (27.0 ± 9.1 vs. 20.0 ± 10.2 U/L, *p =* 0.048), and GGT (41.2 ± 10.1 vs. 34.6 ± 8.2 U/L, *p =* 0.045). Total bilirubin was also significantly higher in the steatosis group (11.2 ± 6.4 vs. 7.0 ± 2.8 µmol/L, *p =* 0.045). The prevalence of hyperuricemia was higher in patients without hepatic steatosis (18.2% vs. 16%, *p =* 0.76), although this difference was not statistically significant. Impaired glucose tolerance or diabetes mellitus was more prevalent in the steatosis group (44.0% vs. 36.4%); however, this was not statistically significant (*p =* 0.29).

## MRI-DERIVED QUANTITATIVE MEASUREMENTS

### Hepatic steatosis

**Figure 1:**
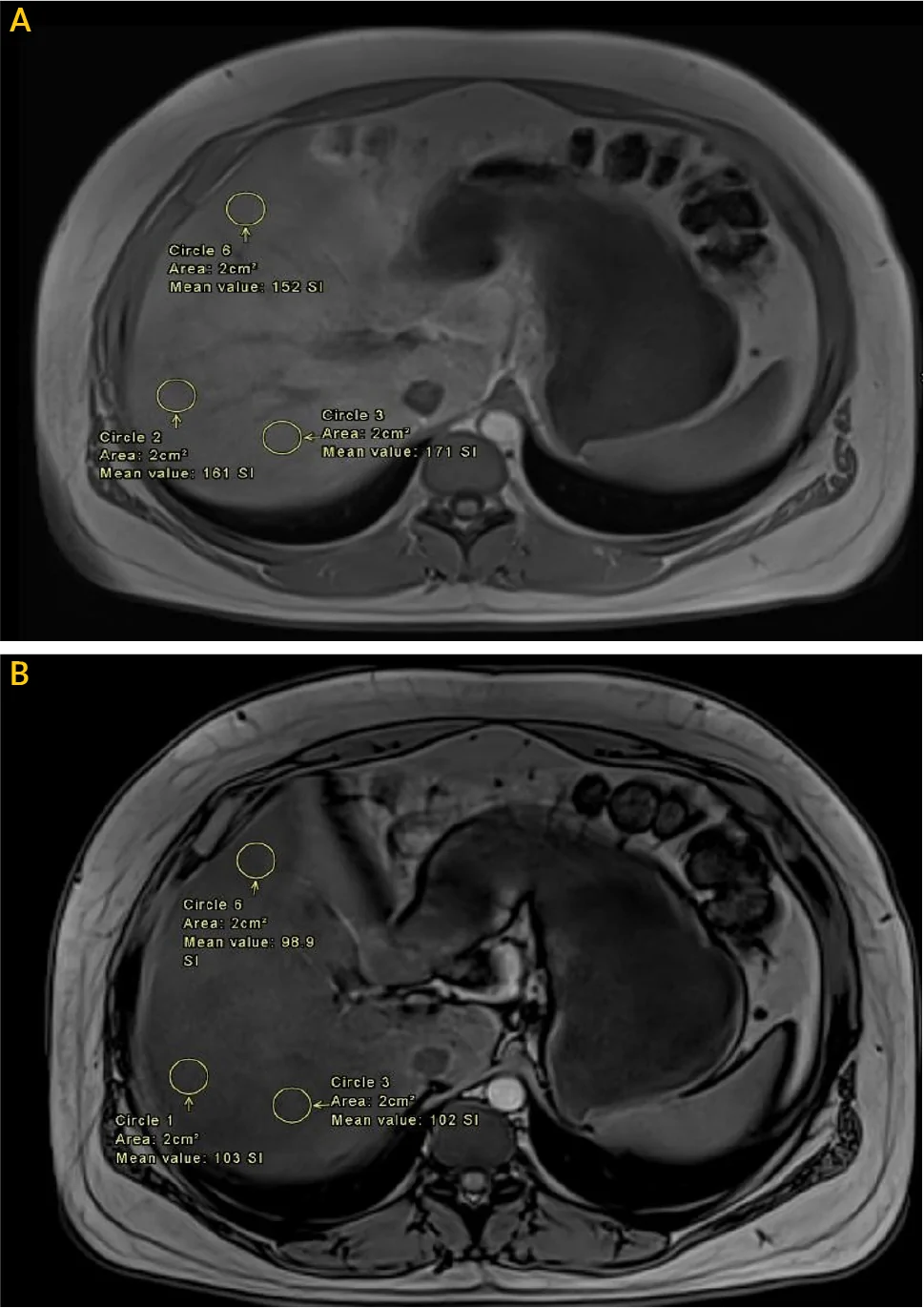
Liver MRI T1-weighted in-phase (**A**) and out-of-phase (**B**) images show diffuse hepatic steatosis, evidenced by a uniform signal drop on the out-of-phase sequence, consistent with diffuse fatty infiltration.

The mean hepatic fat fraction was significantly higher in patients with MRI-defined steatosis compared to those without (45.0 ± 10.1% vs. 5.40 ± 1.51%, *p <* 0.001). The hepatic fat fraction, defined as the signal-intensity ratio between opposed-phase and in-phase images and expressed as a percentage, was significantly lower in the steatosis group, reflecting greater intracellular lipid content (**Table 1** and **Figure 1**).

### Visceral fat

**Figure 2:**
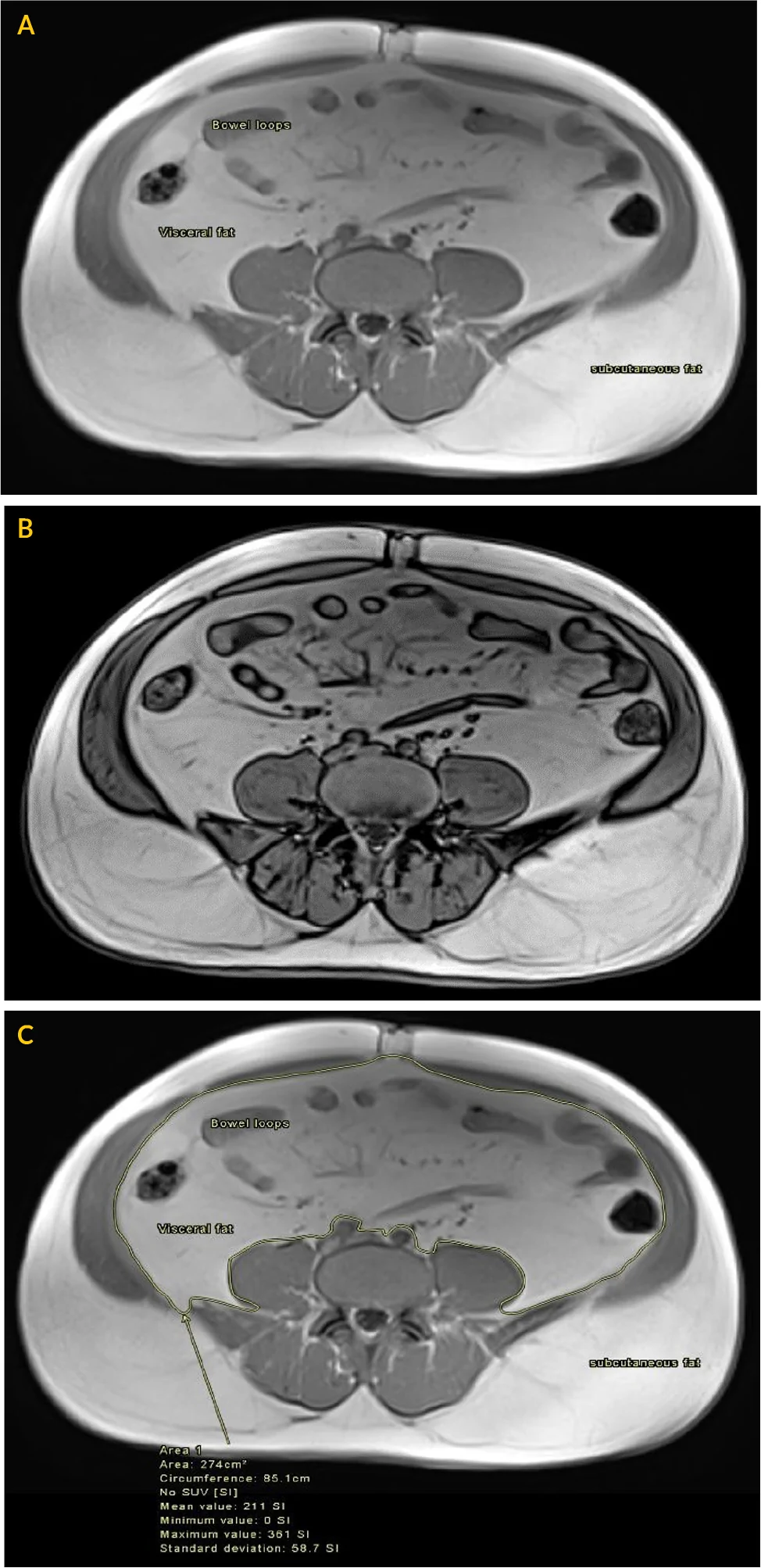
Axial abdominal MRI at the L4-L5 level, depicting visceral fat assessment. (**A**) In-phase T1-weighted image, (**B**) out-of-phase T1-weighted image, and (**C**) in-phase T1-weighted image with segmented visceral fat. The region of interest (ROI) was manually delineated along the abdominal wall (yellow line) to distinguish visceral fat from subcutaneous fat, and the visceral fat area within the ROI was quantified.

**Table 1** and **Figure 2** demonstrate that visceral fat area at the L4-L5 level was significantly greater in patients with hepatic steatosis (35.0 ± 8.0 vs. 23.9 ± 6.7 cm^2^, *p <* 0.001).

### Correlation analyses

**Figure 3:**
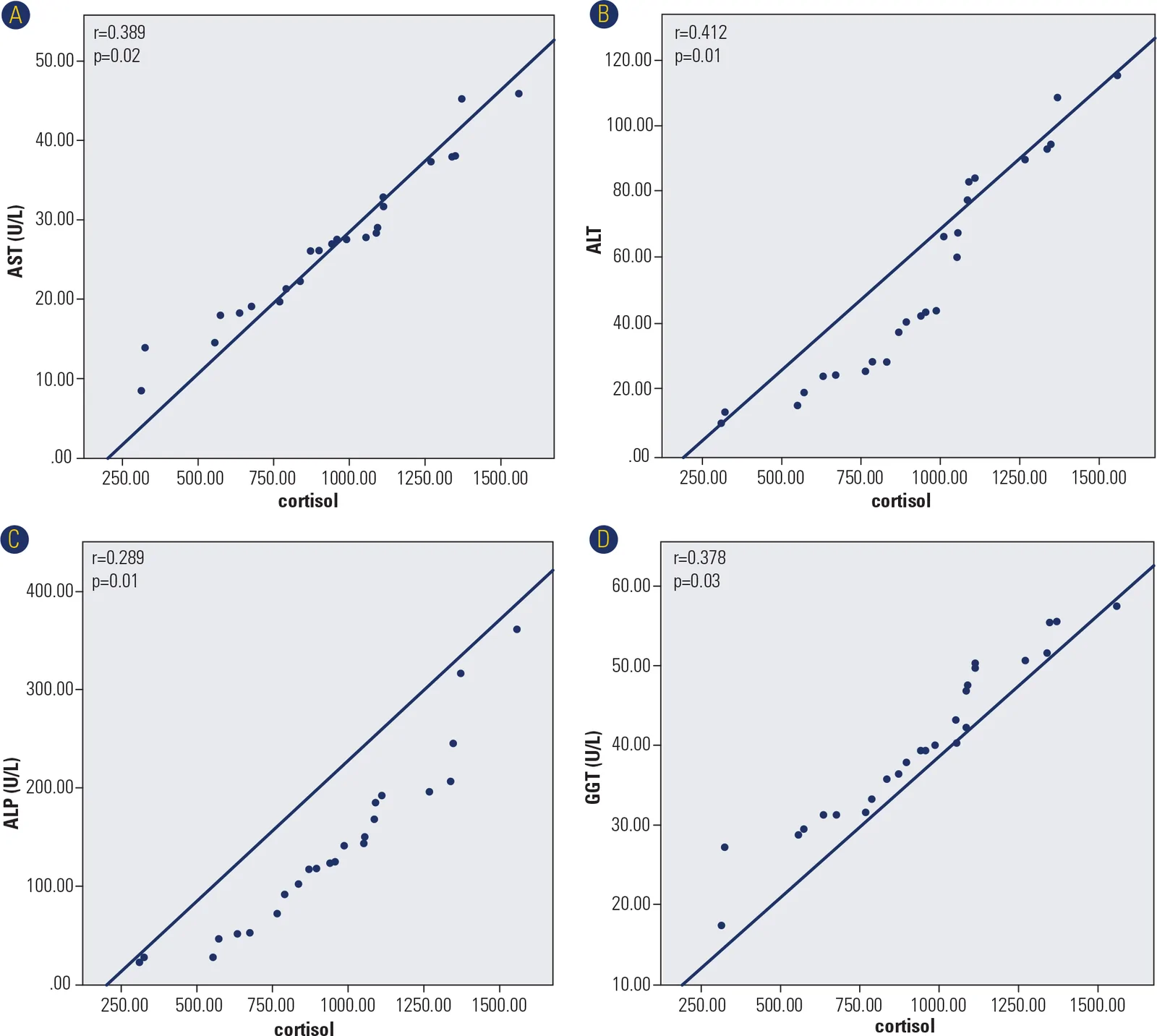
(**A**) Association between serum cortisol and AST. (**B**) Association between serum cortisol and ALT. (**C**) Association between serum cortisol and ALP. (**D**) Association between serum cortisol and GGT.

**Figure 4:**
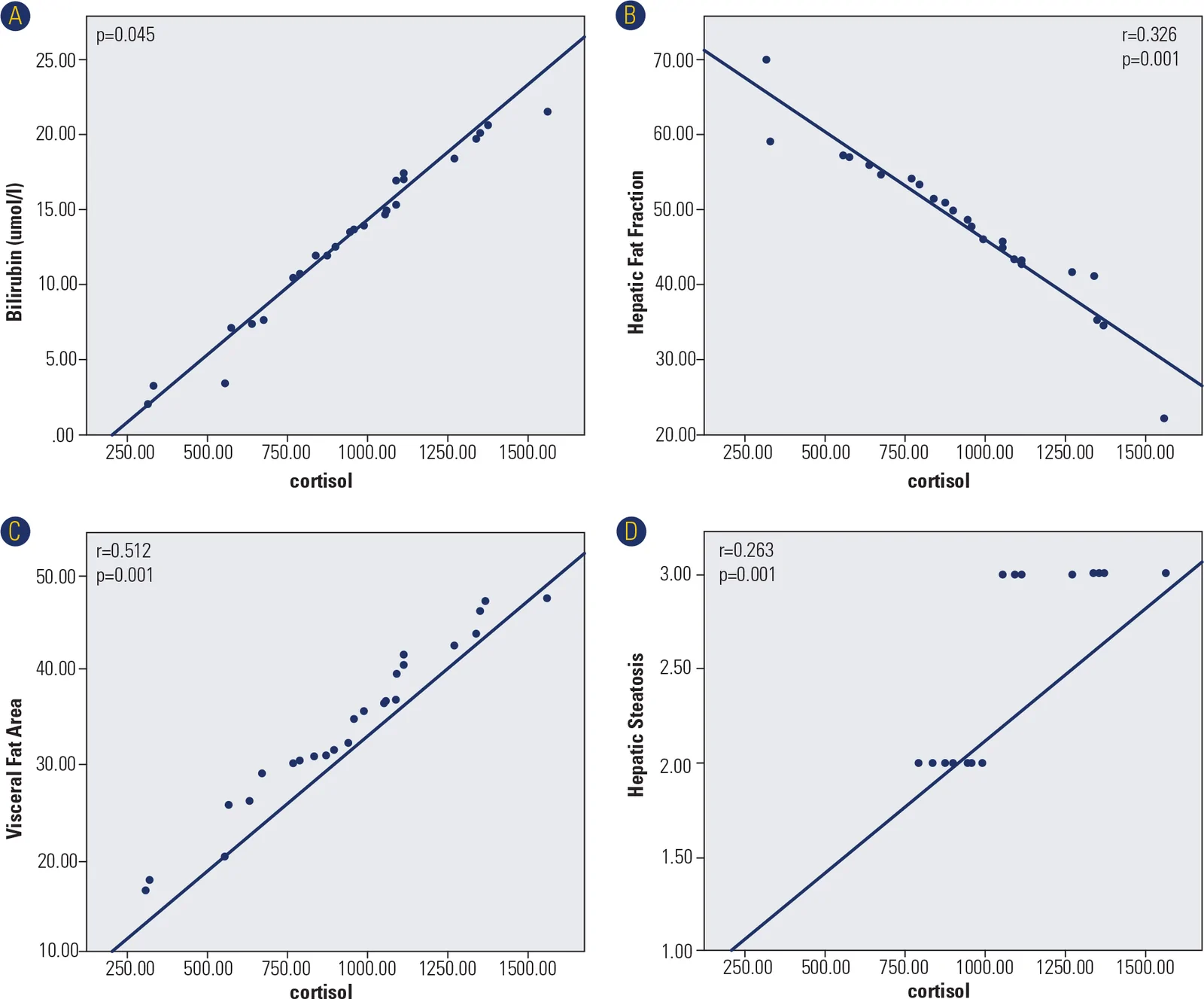
(**A**) Association between serum cortisol and bilirubin. (**B**) Association between serum cortisol and the degree of hepatic attenuation. (**C**) Association between serum cortisol and visceral fat distribution. (**D**) Association between serum cortisol and the degree of hepatic steatosis.

**Figure 3** demonstrated that serum cortisol showed a significant positive correlation with markers of hepatocellular injury, including AST (*r =* 0.389, *p =* 0.02), ALT (*r =* 0.412, *p =* 0.01), ALP (*r =* 0.289, *p =* 0.01), and GGT (*r =* 0.378, *p =* 0.03). Serum cortisol concentration also correlated positively with hepatic fat fraction (*r =* 0.326, *p =* 0.001) (**Figure 4A**), visceral fat area (*r =* 0.512, *p =* 0.001) (**Figure 4B**), and degree of hepatic steatosis (*r =* 0.263, *p =* 0.001) (**Figure 4C**). There was a significant negative correlation between the hepatic signal intensity index and cortisol levels (*r* = -0.424, *p* = 0.01), indicating that higher cortisol levels were associated with greater visceral adiposity and higher hepatic fat content. A strong, statistically significant positive correlation was observed between visceral fat area and the degree of hepatic steatosis (*r =* 0.601, *p <* 0.001). This finding represents one of the principal observations of this study, demonstrating a close quantitative relationship between these two glucocorticoid-sensitive fat depots.

## DISCUSSION

In CS, fat tends to accumulate preferentially in visceral and periorgan compartments rather than subcutaneous tissue, leading to hypertrophic, inflamed VAT with dysregulated adipokines, enhanced lipolysis, and reduced storage capacity. This state is associated with ectopic fat deposition in the liver and other organs. Independent of BMI, active CS is marked by increased waist circumference and remodeling of inflammatory VAT macrophages, indicating qualitative changes in VAT beyond simple expansion (^17^).

Conceptual models position hepatic steatosis as one of several ectopic fat depots closely linked to visceral adiposity, with intrahepatic triglyceride content correlating more strongly with visceral than subcutaneous fat, even among patients evaluated for adrenal disease. In CS, cortisol-induced visceral obesity, insulin resistance, and atherogenic dyslipidemia are associated with hepatic triglyceride accumulation due to increased de novo lipogenesis, higher free fatty acid flux from visceral fat, and reduced very low density lipoprotein export. Recent evidence indicates that hypercortisolism is linked to ectopic fat deposition in the pancreas, renal sinus, and skeletal muscle, further supporting CS as a clinical model of systemic ectopic lipid overload, with liver fat as a central feature of this phenotype (^18^).

Hepatic steatosis was highly prevalent in this cohort, with chemical shift-encoded MRI demonstrating MASLD in 69.4% of patients with active, treatment-naïve CS. This proportion is more than double that reported in the general Egyptian population (31.6%), and clearly exceeds global estimates for MASLD in unselected populations (25%-30%) (^19^,^20^), indicating that liver fat accumulation represents a core metabolic feature of active CS rather than an incidental co-morbidity. Notably, the 69.4% prevalence observed here substantially surpasses the 20% reported by Rockall and cols. (^9^) among 50 CS patients, highlighting how advances in imaging and differences in study design can markedly affect the perceived disease burden.

Several methodological and demographic factors may account for these discrepancies. Foremost, earlier work relied on CT-based liver fat estimation, which infers steatosis from hepatic attenuation (Hounsfield units) and poorly detects mild-to-moderate fat accumulation (<30% histological steatosis), in addition to being susceptible to confounding by hepatic iron, edema, or glycogen (^10^). In contrast, chemical-shift-encoded MRI leverages the intrinsic resonance-frequency differences between water and fat protons to provide sensitive, quantitative fat-fraction measurements across the clinically relevant range, thereby capturing subclinical and early-stage steatosis that CT may miss. In addition, this study deliberately included only patients with active, untreated CS at the time of imaging, ensuring ongoing exposure to chronic hypercortisolism. Earlier studies may have inadvertently enrolled individuals with partially treated disease or intermittent cortisol excess, potentially underestimating the true prevalence of steatosis. It is also plausible that the Egyptian population has a higher baseline susceptibility to hepatic steatosis due to genetic background, dietary habits, or clustering of metabolic risk factors, as suggested by Tomah and cols. (^19^), further amplifying the effect of glucocorticoid excess on liver fat accumulation.

Our findings are consistent with the recent work of Yu and cols. (^21^), who used proton magnetic resonance spectroscopy, the most sensitive noninvasive technique for hepatic fat quantification, to study 48 CS patients and 30 individuals with non-functioning adrenal incidentalomas. They reported a 66% prevalence of hepatic steatosis in the CS group, closely mirroring our 69.4%. The convergence of these results, despite differences in geography, patient selection, and MRI technique (spectroscopy versus chemical shift-encoded imaging), provides compelling evidence that hepatic steatosis is extremely common in active CS and likely under-recognized when less sensitive modalities are used.

In this current study, an external eucortisolemic control cohort matched for age, sex, BMI, or metabolic comorbidities was not included. These factors may substantially influence visceral adiposity and hepatic fat accumulation and therefore represent potential confounders. However, despite these limitations, significant associations remained among serum cortisol levels, visceral fat area, and hepatic steatosis within the active CS cohort, suggesting a biologically meaningful relationship between hypercortisolism and ectopic fat deposition.

Mechanistically, the relationship between hypercortisolism and hepatic steatosis is biologically plausible and appears to arise through direct and indirect pathways. Indirectly, chronic glucocorticoid excess preferentially expands and metabolically disrupts visceral adipose tissue, promoting increased lipolysis and reduced insulin sensitivity in these depots. As a result, the portal circulation is exposed to increased free fatty acid flux, thereby delivering more lipid substrate to hepatocytes and promoting intrahepatic triglyceride synthesis and storage. The strong positive correlation observed between visceral fat area and steatosis severity (*r =* 0.601, *p <* 0.001) is consistent with this portal “lipid spillover” model. Cortisol also acts directly on hepatocytes via GRs, upregulating key lipogenic enzymes such as fatty acid synthase and acetyl-CoA carboxylase, while simultaneously suppressing PPARα-driven fatty acid oxidation. Local regeneration of active cortisol within the liver by 11β-hydroxysteroid dehydrogenase type 1 further amplifies these effects by enhancing intrahepatic glucocorticoid signaling, independent of circulating cortisol concentrations (^22^). Collectively, these indirect (visceral adiposity-mediated) and direct (hepatocyte-intrinsic) mechanisms create a metabolic milieu that strongly favors hepatic fat accumulation, providing a coherent pathophysiological explanation for the high burden of MASLD observed in active CS.

The finding that roughly 70% of patients with active CS have MRI-detectable hepatic steatosis has clear clinical implications. This suggests that MASLD screening should be considered as part of the metabolic evaluation in newly diagnosed CS, utilizing the same chemical shift-encoded MRI sequences already obtained for adrenal imaging to allow concurrent liver fat quantification without additional scan time or radiation exposure. Nevertheless, the present cross-sectional design does not determine whether MRI-derived hepatic or visceral fat measurements independently predict clinical outcomes or improve risk stratification beyond standard metabolic markers.

The relatively high prevalence of hepatic steatosis reported in this cohort should be interpreted with caution. As this study was conducted at a single center in Egypt, where metabolic dysfunction and hepatic steatosis are known to be relatively high, population-specific genetic, environmental, dietary, and lifestyle factors may have contributed to the findings. Therefore, the reported prevalence estimates may not be directly generalizable to other ethnic or geographic populations, and confirmation in multicenter studies with more diverse cohorts is required.

Identification of steatosis is crucial, as MASLD is associated with increased cardiovascular mortality, progression to steatohepatitis, and, in some patients, fibrosis and cirrhosis. In this cohort, CS patients with hepatic steatosis exhibited higher cortisol levels, more atherogenic lipid profiles, elevated liver enzymes, and greater visceral adiposity, indicating a particularly high-risk cardiometabolic phenotype. These findings support the need for intensified management that normalizes cortisol excess and aggressively addresses cardiometabolic risk factors to reduce long-term hepatic and cardiovascular complications.

Although increased visceral and hepatic fat may contribute to adverse cardiometabolic outcomes in active CS, this cross-sectional study cannot determine whether these MRI-derived abnormalities independently predict clinical outcomes or improve risk stratification beyond established metabolic markers.

The main strength of this study is its precise quantification of adiposity. To our knowledge, this is the first CS cohort in which dedicated semi-automated software (SliceOmatic) was used to obtain reproducible, MRI-based measurements of visceral fat area, reducing operator dependence and providing continuous quantitative data that enhance statistical power and comparability.

Several limitations should be acknowledged. Adipose tissue distribution and hepatic steatosis in CS are likely influenced not only by cortisol excess, but also by disease duration, chronicity, severity of hypercortisolism, concomitant metabolic disorders, medication exposure, physical activity, and hormonal status, including menopause. Several of these variables were incompletely captured in this retrospective cohort, so residual confounding cannot be excluded. The absence of a matched eucortisolemic control group limited the ability to determine the independent contribution of cortisol excess relative to other determinants of visceral adiposity and hepatic steatosis. The relatively small cohort size, although comparable to prior MRI-based studies in rare endocrine disorders, may have limited statistical power and restricted the ability to perform comprehensive multivariable or subgroup comparisons. Despite the modest cohort size, several statistically significant associations were observed, including correlations between cortisol levels, visceral adiposity, and hepatic steatosis severity, suggesting that the detected relationships are biologically robust.

Although intra-observer reproducibility was assessed by repeat analysis of 10 examinations after a 4-week interval, quantitative reproducibility metrics such as the intraclass correlation coefficient and coefficient of variation were not reported; we acknowledge that inclusion of these metrics would further strengthen assessment of measurement reliability.

Larger multicenter, prospective studies are required to validate and extend these findings. The absence of a eucortisolemic control group with similar metabolic risk profiles limits the ability to attribute the high steatosis burden specifically to glucocorticoid excess. Interpretation of the observed prevalence of steatosis and group comparisons should be considered in light of these limitations. First, no age-, sex-, or BMI-matched eucortisolemic control group was included, limiting the ability to determine whether the high prevalence of hepatic steatosis observed is independently attributable to hypercortisolism rather than to metabolic risk factors. Second, patients with and without steatosis differed significantly in age and BMI, which are established determinants of hepatic fat and visceral adiposity. Therefore, these differences may have contributed to group differences in hepatic fat content. The cross-sectional design and limited sample size also necessitate that observed associations be interpreted with caution and not as evidence of a causal or independent effect of cortisol excess. Larger, controlled studies with multivariable adjustment are required to clarify the relative contributions of hypercortisolism and other metabolic risk factors to ectopic fat accumulation in CS. The absence of liver biopsy precludes assessment of steatohepatitis and fibrosis, which are key prognostic markers, and inter-observer variability in MRI fat measurements was not examined. Finally, the single-center design limits generalizability, underscoring the need for larger, multicenter studies in more diverse populations.

Although MRI offers a highly sensitive, quantitative assessment of hepatic fat content without exposure to ionizing radiation, CT remains an important and widely used modality in clinical practice. CT-based assessment of visceral adiposity is extensively validated, broadly available, relatively inexpensive, and can often be performed using scans obtained for other indications without additional imaging. Therefore, the choice between MRI and CT should be guided by clinical context, imaging resource availability, and specific research or diagnostic objectives. While MRI may offer greater sensitivity for detecting mild hepatic fat accumulation, CT remains a practical and clinically valuable tool for body composition assessment in many settings.

In conclusion, hepatic steatosis consistent with MASLD was detectable in approximately 70% of patients with active, treatment-naïve CS when evaluated using sensitive, quantitative MRI-based fat mapping. This burden clearly exceeds prior CT-derived estimates. The severity of liver fat accumulation showed a robust association with both circulating cortisol levels and MRI derived visceral fat area. Because abdominal MRI is routinely performed for adrenal characterization in CS, leveraging the same examination to quantify hepatic and visceral fat provides a high-yield, radiation-free approach for integrated metabolic risk assessment. These findings support systematic incorporation of hepatic fat quantification into the baseline imaging workup for patients with newly diagnosed CS. Although our findings demonstrate significant associations among hypercortisolism, visceral adiposity, and hepatic steatosis in active CS, the small sample size and absence of a matched eucortisolemic control group limit the ability to determine the independent impact of cortisol excess. Larger, prospective, controlled studies are needed to validate these findings and clarify the relative contributions of hypercortisolism and other metabolic risk factors to ectopic fat accumulation.

### Future research avenues

Prospective longitudinal studies with larger, multicenter cohorts are required to dete rmine the prognostic significance, therapeutic responsiveness, and clinical utility of MRI-derived quantification of hepatic and visceral fat in CS. These studies should evaluate whether effective surgical or medical control of cortisol excess results in meaningful regression of liver fat and visceral adipose tissue, and whether baseline hepatic steatosis identifies patients at risk for persistent metabolic dysfunction despite biochemical remission. Incorporation of advanced MRI techniques, such as MR elastography for noninvasive fibrosis staging and multiparametric protocols integrating fat, iron, and tissue stiffness mapping, would substantially enhance the depth of phenotyping in future research. Given the single-center design and population-specific characteristics of the current study, the reported prevalence should be interpreted with caution and not directly extrapolated to other populations. Further multicenter studies across different ethnic and geographic settings are needed to confirm the generalizability of these findings.

**Ethical approval and consent to participate:** this study was approved by the Ethics Committee of the Faculty of Medicine, Alexandria University (protocol code 0306946) and conducted in accordance with the principles of the Declaration of Helsinki. Written informed consent was obtained from all participants.

## Data Availability

datasets related to this article will be available upon request to the corresponding author.
